# The complete mitochondrial genome of *Acanthopagrus pacificus* (Perciformes, Sparidae) from Iriomotejima Island, Okinawa, Japan

**DOI:** 10.1080/23802359.2022.2080606

**Published:** 2022-06-15

**Authors:** Kentaro Kawai, Hiroki Fujita, Tetsuya Umino

**Affiliations:** Graduate School of Integrated Sciences for Life, Hiroshima University, Higashi-Hiroshima, Hiroshima, Japan

**Keywords:** Mitochondrial DNA, next generation sequencing, Pacific seabream, phylogeny, Sparid

## Abstract

The Pacific seabream, *Acanthopagrus pacificus*, is a commercially valuable resource for fisheries around the West Pacific Ocean countries. Although the phylogenetic position of the *A. pacificus* within the genus *Acanthopagrus* changes by the differences in the target gene regions or fish sampling locations, the genetic information of the species is quite limited. In this study, the complete mitochondrial genome of *A. pacificus* from Iriomotejima Island, Okinawa, Japan was determined. The sequence is 16,640 bp in length and consists of 13 protein-coding genes, 2 rRNA genes (12S rRNA and 16S rRNA), 22 tRNA genes, and one control region. A reconstructed phylogenetic tree showed that *A. pacificus* has the furthest relationship to other *Acanthopagrus* species in the genus.

Mitochondrial genomes have been used for the study of species evolution, phylogenetic relationships, and population structure because of their small genome size, rapid evolutionary rates, and absence of sequence recombination. In phylogenetic analyses, the resolution and support were improved by using the whole mitochondrial genome than using protein-coded regions (Cameron et al. 2007). Recent phylogenetic studies using a complete mitochondrial genome revealed detailed evolutionary history in some taxon (Nardi et al. 2010: Ma et al. 2015; Wang et al. 2016).

Genus *Acanthopagrus* species are commercially and recreationally important coastal fishes from temperate to the tropical zone (Tseng et al. 2009; Iwatsuki and Heemstra 2011; Kawai et al. 2020). Within the genus, *Acanthopagrus schlegelii* and *Acanthopagrus latus*, targeted species for stock enhancement and aquaculture in Asian countries, are studied intensively not only in reproductive biology (Law and Sadovy de Mitcheson 2017; Wang et al. 2020; Kawai et al. 2021) but also in genetic population structure (Song et al. 2021; Yamashita et al. 2021; Hsu et al. 2022). Same as the above two species, the Pacific seabream, *Acanthopagrus pacificus* Iwatsuki et al. 2010, is a commercially important species in the West Pacific Ocean (Iwatsuki et al. 2010; Tran et al. 2021), although information on the population genetics of the species has not been reported. In addition, although partial sequences analysis of mitochondrial DNA and AFLP (amplified fragment length polymorphism) analysis of nuclear DNA indicated some ideas of the phylogenetic position of the *A. pacificus* within the genus *Acanthopagrus*, these results varied with the differences in the target regions or collected locations of fish (Hsu et al. 2011; Wu et al. 2018). Because *A. pacificus* distributes widely in the West Pacific Ocean (Iriomote Island in Okinawa of Japan, Taiwan, southern China, Viet Nam, Philippines, Thailand, Malaysia, Indonesia, Papua New Guinea, northern Australia) (Iwatsuki et al. 2010), the species have a possibility to include cryptic species, as a similar situation of *Acanthopagrus morrisoni* separated from *A. latus* complex (Iwasaki et al. 2013). Here, we determined the complete mitochondrial genome sequence of *A. pacificus* from Iriomotejima Island, Okinawa Prefecture, Japan. The results of this study will provide reference not only for the resolution of the complexed phylogeny of the genus *Acanthopagrus* but also for the genetic population research of *A. pacificus*.

The experiments in this study complied with the fundamental guidelines for the proper conduct of animal experiments and related activities in academic research institutions under the jurisdiction of the Ministry of Education, Culture, Sports, Science, and Technology (Notice No. 71, 1 June 2006). The *A. pacificus* specimen was collected by line fishing at Urauchi River (24.400783 N, 123.782696 E) in Iriomotejima Island, Okinawa, Japan, on 7 March 2022. The specimen was deposited at Hiroshima University Museum (Deposit No. HUM-I-02335: Norio Shimizu, museum@hiroshima-u.ac.jp). The specimen was identified based on the description of key characteristics of the *Acanthopagrus* species (Iwatsuki 2013). Total genomic DNA was extracted from the pectoral fin by the phenol-chloroform Isoamyl alcohol extraction method. The high-throughput DNA sequencing was performed by Bioengineering Lab. Co., Ltd. (Sagamihara, Japan) using DNBSEQ-G400 system (MGI Tech Co., Ltd.: Shenzhen, China) with a paired-end 200 bp sequencing. The obtained raw reads (5,916,726 reads, 2,366,690,400 bp) were trimmed using fastp (Chen et al. 2018) to remove adapters and low-quality reads. After filtering, the paired-end reads were assembled to a complete mitochondrial genome using NOVOplasty (Dierckxsens et al. 2017), with A. *pacificus* (LC458140) as the seed reference. Mitochondrial genes were annotated using MitoAnnotator (Iwasaki et al. 2013).

The complete mitogenome of *A. pacificus* was 16,640 bp in length (GenBank accession no. LC707238) and consisted of 13 protein-coding genes, 2 rRNA genes (12S rRNA and 16S rRNA), 22 tRNA genes, and one control region. The gene arrangement was identical to that observed in *A. schlegelii* (Shi et al. 2012; Ma et al. 2016) and *A. latus* (Xia et al. 2008; Pan et al. 2021).

Phylogenetic analysis of genus *Acanthopagrus* was conducted with the complete mitochondrial genome sequences of *A. schlegelii* (accession no. JQ746035, KT805958 and LC680889) and *A. latus* (accession no. EF506764 and MN909968) obtained from NCBI (Xia et al. 2008; Shi et al. 2012; Ma et al. 2016; Pan et al. 2021). The Complete mitochondrial genome of *Rhabdosargus sarba* (accession no. KM272585) (Li et al. 2016) from NCBI served as an outgroup. These sequences were aligned using MUSCLE (Edgar 2004). The phylogenetic tree was reconstructed using MEGA X (Kumar et al. 2018) with the maximum likelihood method (K2P + G + I model). Bootstrap analyses for the phylogenetic tree were conducted with 1000 replications (Figure 1).

**Figure 1.**
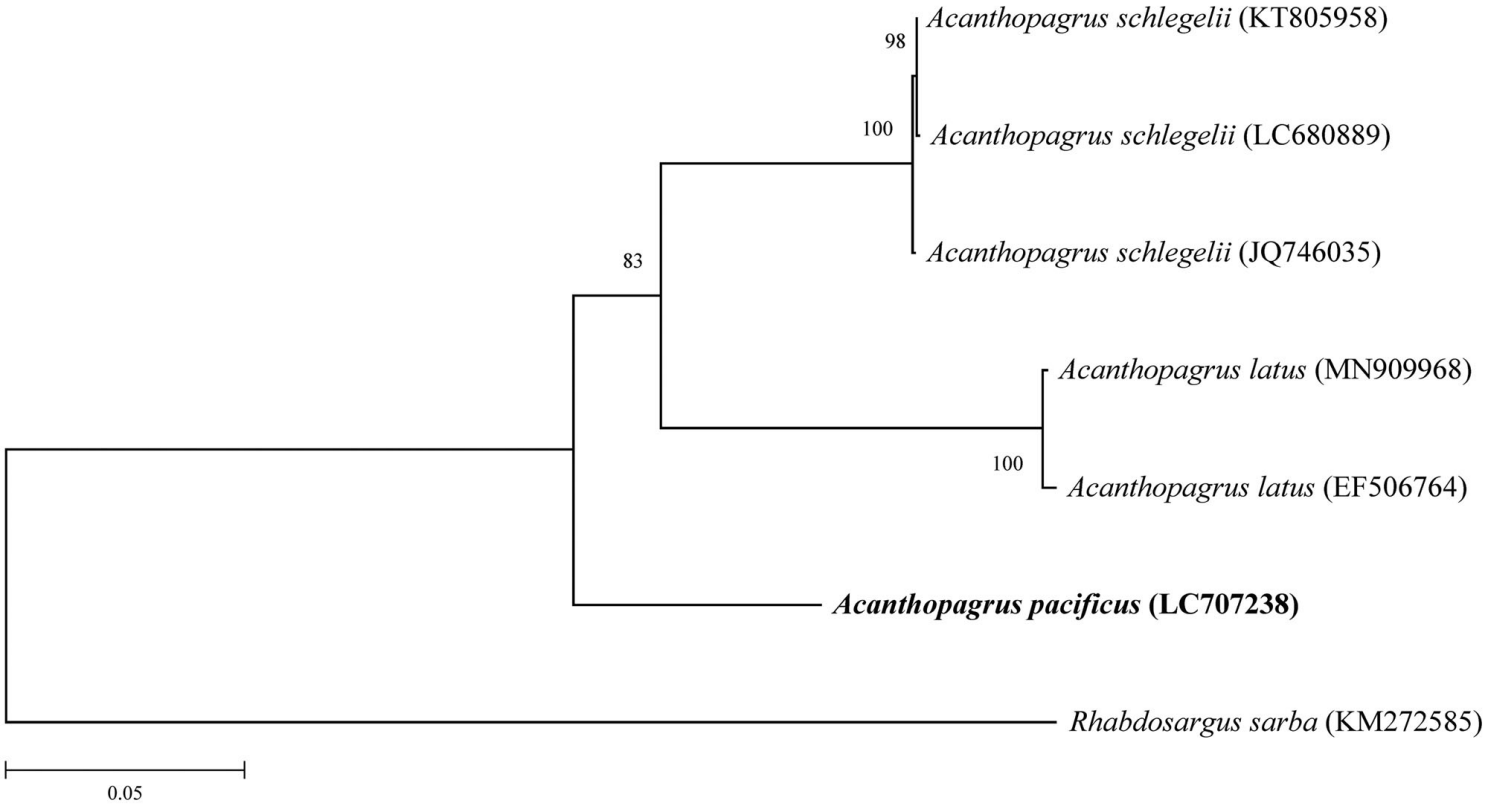
A phylogenetic tree reconstructed with complete mitochondrial genome sequences of *Acanthopagrus pacificus* and other species.

The phylogenetic tree showed that *A. pacificus* has the furthest relationship to the *A. latus* and *A. schlegelii*, which supports the previous studies on phylogenetic relationships of *Acanthopagurus* species using partial mitochondrial genome sequences and nuclear DNA AFLP analysis (Hsu et al. 2011).

## Data Availability

The mitochondrion genome sequence data that support the findings of this study are openly available in GenBank with the accession no. LC707238 (https://www.ncbi.nlm.nih.gov/nuccore/LC707238). The associated BioProject, DRA, and BioSample numbers are PRJDB13470, DRA013995, and SAMD00468474 respectively.
